# Higher CD19+CD25^+^ Bregs are independently associated with better graft function in renal transplant recipients

**DOI:** 10.1186/s12882-021-02374-2

**Published:** 2021-05-17

**Authors:** Eman H. Ibrahim, Mostafa G. Aly, Gerhard Opelz, Christian Morath, Martin Zeier, Caner Süsal, Douaa M. Sayed, Eman Hassan, Naruemol Ekpoom, Volker Daniel

**Affiliations:** 1 Transplantation Immunology, Institute of Immunology, University Hospital Heidelberg, Im Neuenheimer Feld 305, 69120 Heidelberg, Germany; 2 Clinical Pathology Department, South Egypt Cancer Institute, Assiut University, Asyut, Egypt; 3 Department of Nephrology, University Hospital Heidelberg, Heidelberg, Germany; 4 Nephrology Unit, Internal Medicine Department, Assiut University, Asyut, Egypt

**Keywords:** Bregs, Renal transplantation, Tregs, GFR

## Abstract

**Background:**

The Identification of B cell subsets with regulatory functions might open the way to new therapeutic strategies in the field of transplantation, which aim to reduce the dose of immunosuppressive drugs and prolong the graft survival. CD25 was proposed as a marker of a B-cell subset with an immunosuppressive action termed Bregs. The effect of CD19 + CD25 + Bregs on graft function in renal transplant recipients has not yet been elucidated. We investigated a potential impact of CD19 + CD25 + Bregs on renal graft function as well as a possible interaction of CD19 + CD25 + Bregs with peripheral Tregs in healthy controls, end-stage kidney disease patients (ESKD), and renal transplant recipients. Moreover, we aimed to investigate the association of CD19 + CD25 + Bregs with serum IL-10, TGF-ß1, and IFN-γ in the same study groups.

**Method:**

Thirty-one healthy controls, ninety renal transplant recipients, and eighteen ESKD patients were enrolled. We evaluated the CD19 + CD25 + Bregs and Treg absolute counts. Next, we investigated CD19 + CD25 + Bregs as predictors of good graft function in multiple regression and ROC analyses. Finally, we evaluated the association between CD19 + CD25+ Bregs and serum IL-10, TGF-ß, and IFN-γ.

**Results:**

ESKD patients and renal transplant recipients showed lower counts of CD19 + CD25+ Bregs compared to healthy controls (*p* < 0.001). Higher CD19 + CD25+ Breg counts were independently associated with a better GFR in renal transplant recipients (unstandardized B coefficient = 9, *p* = 0.02). In these patients, higher CD19 + CD25+ Bregs were independently associated with higher Treg counts (unstandardized B = 2.8, *p* = 0.004). In ROC analysis, cut-offs for CD19 + CD25 + Breg counts and serum TGF-ß1 of 0.12 cell/μl and 19,635.4 pg/ml, respectively, were shown to provide a good sensitivity and specificity in identifying GFR ≥ 30 ml/min (AUC = 0.67, sensitivity 77%, specificity 43%; AUC = 0.65, sensitivity 81%, specificity 50%, respectively). Finally, a significant positive association between CD19 + CD25+ Bregs and TGF-ß1 was shown in renal transplant recipients (*r* = 0.255, *p* = 0.015).

**Conclusions:**

Our findings indicate that higher counts of CD19 + CD25+ Bregs are independently associated with better renal function and higher absolute Treg counts in renal transplant recipients.

**Supplementary Information:**

The online version contains supplementary material available at 10.1186/s12882-021-02374-2.

## Background

B cells have long been known for antibody production and their function as antigen presenting cells [1]. B regulatory cells (Breg) represent a relatively recent topic in the literature. B cell subsets with a potential regulatory function on other immune cells were first described in the 1970s [2, 3]. An inhibitory role of B cells on T cell activation and proliferation has been suggested upon activation of a delayed type hypersensitivity after administration of cyclophosphamide, a B cell depleting agent [2, 3]. Intriguingly, and supporting a regulatory function of some B cell subsets, a study showed that B cell deficiency in mice with multiple sclerosis (MS) led to an absence of spontaneous recovery from artificial autoimmune encephalitis [4]. Since then, many research efforts have been directed at identifying the phenotype and the mechanisms through which Bregs exert their function [5–11]. Different Breg phenotypes have been identified and studied in association with different diseases, such as CD19 + CD24highCD27+^,^ CD19^+^IgM + CD27+ and CD19 + CD1dhighCD5+ in autoimmune diseases [12–14], CD19 + CD38 + CD1d + IgM + CD147+ and CD24highCD27+ in cancer [15–17], CD19 + CD38+ and CD19 + CD24highCD38high in viral and bacterial diseases [9, 18, 19], CD19 + CD1d + CD5+, CD19 + IgM + IgD+ and CD19 + CD5 + IL10+ in parasitic diseases [20–22], and CD19 + CD24highCD38high, CD19 + IL10+ and CD19 + IgM + CD27+ in transplant recipients [12, 23–25].

The expression of CD25 on B cells has been proposed as a one of the markers of Bregs in humans [26]. In vitro experiments proved a clear regulatory role of CD19 + CD25+ Bregs. Kessel et al. reported a dose-dependent inhibitory effect of CD19 + CD25 + Bregs on stimulated CD4+ T cells as well as a stimulatory effect of CD19 + CD25 + Bregs on Foxp3 and CTLA-4 expression in Tregs. The effect of CD19 + CD25 + Bregs on Tregs was mediated through direct contact and TGF-β [26]. Although the immunoregulatory role and the mechanism of action of CD19 + CD25+ Bregs have been delineated in vitro, the role of these cells in renal transplant recipients in terms of their contribution to a good graft function has not yet been elucidated. The primary objective of the current study was to show whether CD19 + CD25+ Bregs might independently impact the glomerular filtration rate (GFR) in renal transplant recipients. As a secondary objective, we aimed to further characterize the CD19 + CD25+ Bregs through their expression of Foxp3. In addition, we aimed to investigate a possible interaction between Bregs and Tregs.

## Methods

### Patients

In the current observational cross-sectional study, 31 healthy blood donors and staff members with normal routine medical check-ups aged between 18 and 60 years served as controls. Samples from 18 end stage kidney disease patients (ESKD) on regular hemodialysis and 90 renal transplant recipients aged 18 years and above from the Department of Nephrology, University of Heidelberg were obtained. Exclusion criteria were as follows: current rejections or previous treatment with rituximab. Patients as well as healthy controls gave written informed consent for the tests performed within this study. The study was approved by the Heidelberg ethical committee (S-225/2014) and conducted in adherence to the Declaration of Helsinki. Table 1 summarizes the demographic data of the patients.

**Table 1:** Demographic data of healthy controls (HC), end-stage renal disease patients (ESRD), and renal transplant recipients (Tx)

	HC (***n*** = 31)	ESRD (***n*** = 18)	Tx (***n*** = 90)
**Age (years, median, IQR)**	43 (35–54)	65 (51–73)	56 (45–63)
**Sex (n, %)**			
**Female**	17 (55)	6 (33)	27 (30)
**Male**	14 (45)	12 (67)	63 (70)
**Days post-transplant (median, IQR)**	–	–	410 (22–3452)
**Type of donor (n, %)**			
**Living**	–	–	17 (19)
**Deceased**	–	–	73 (81)
**Graft No. (n, %)**			
**First**	–	–	82 (91)
**Second**	–	–	7 (8)
**Third**	–	–	1 (1)
**Delayed graft function (n, %)**	–	–	9 (10)
**Cold ischemia time (min, median, IQR)**	–	–	765 (485–984)
**GFR ml/min (median, IQR)**	–	–	40 (24–55)
**Serum creatinine (median, IQR)**	–	–	1.8 (1.3–2.8)
**Previous CMV infection**	–	–	23 (26)
**Previous BKVN (n, %)**	–	–	3 (3)
**Patients with previous rejection (n, %)**			
**Yes**	–	–	26 (29)
**No**	–	–	64 (71)
**Protein/creatinine ratio g/mol (median, IQR)**	–	–	24 (13–48)
**CRP mg/l (median, IQR)**	–	–	10 (2–26)
**Etiology of the end stage renal disease (n, %)**			
**Chronic glomerulonephritis**	–	6 (33)	32 (36)
**Diabetes**	–	4 (22)	11 (12)
**Hypertension/ischemic**	–	–	4 (4)
**ADPKD**	–	2 (11)	10 (11)
**Hereditary/ congenital**	–	1 (6)	8 (9)
**Others**	–	3 (17)	15 (17)
**Unknown**	–	2 (11)	10 (11)
**Induction immunosuppression (number and percentage)**			
**Basiliximab**			77 (85.6)
**ATG**			13 (14.4)
**Maintenance immunosuppression (number and percentage of patients)**			
**Ciclosporine**	–	–	41 (46)
**Tacrolimus**	–	–	44 (49)
**MMF**	–	–	75 (83)
**Steroids**	–	–	86 (96)
**Everolimus**	–	–	3 (3)
**Azathioprine**	–	–	1 (1)

*Abbreviations*: *HC* Healthy control, *ESRD* End-stage renal disease, *Tx* Transplant recipients, *IQR* Interquartile range, *CMV* Cytomegalovirus, *BKVN* Polyoma virus nephropathy, *GFR* Glomerular filtration rate, *ADPKD* Autosomal dominant polycystic kidney disease, *MMF* Mycophenolate mofetil

### Determination of lymphocyte subsets

For identification of lymphocyte subsets, 50 μl of whole blood was incubated with fluorochrome-labeled monoclonal antibodies against CD3, CD4, CD8, CD45, (Cat. no. 342417) CD19, and CD16 + CD56 (Cat. no 342416) (all from BD Biosciences). After vortexing and incubation in the dark at room temperature for 15 min, 500 μl BD FACS Lyse solution (1:10) was added. The tubes were then vortexed and incubated in the dark at room temperature for 15 min. Finally, all samples were evaluated with four-color FACSCalibur II double-laser flow cytometer (BD Biosciences). At least 100,000 events were analyzed in the initial FSC/SSC dot plot.

### Determination of CD19 + CD25+ Bregs and Tregs

**Fig. 1:**
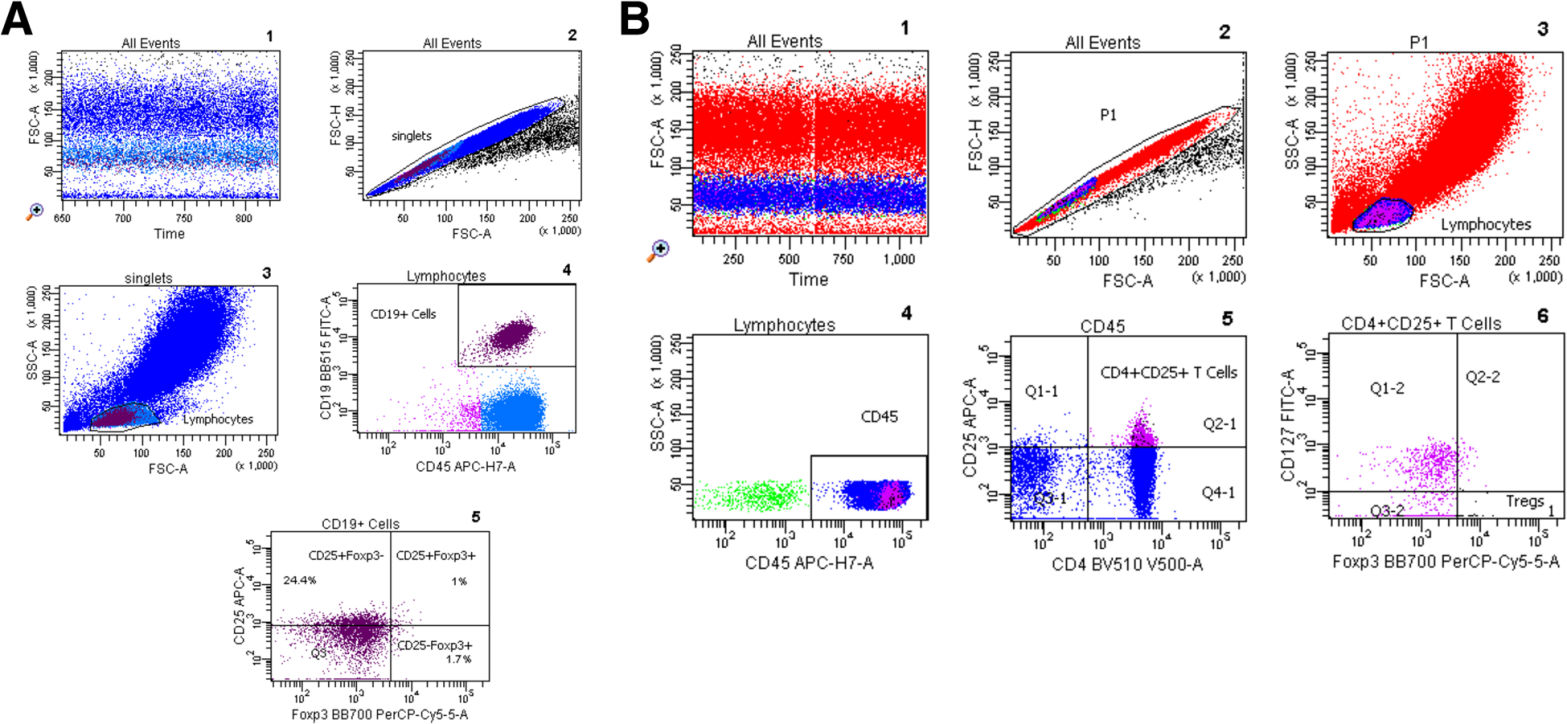
**a** Gating strategy for CD19 + CD25+ Bregs. (1) After ensuring the stability of the run and (2) exclusion of doublets, (3) lymphocytes were gated according to size. (4) Subsequently, CD19 was plotted versus CD45 to identify CD19+ cells. CD25 was then plotted against Foxp3 to further characterize CD19 + CD25+ Bregs into Foxp3- and Foxp3 + . **b** Gating strategy for Tregs. All the events were analyzed after (1) ensuring stability of the run and (2) exclusion of doublets. (3) Lymphocytes were gated according to size. (4) Afterwards, CD45 was gated to focus on lymphocytes. (5) CD25 vs CD4 dot plot allows the identification of CD4 + CD25+ T lymphocytes, (6) Foxp3 + CD127- cells were gated out of CD4 + CD25+ lymphocytes to identify Tregs

Flow cytometric determinations were performed immediately after arrival of the blood samples in the lab. Fluorochrome-labeled monoclonal antibody against CD45 (5 μl; BD cat. no. 560178), CD19 (5 μl; BD cat. no. 564456), CD25 (20 μl; BD cat. no. 555434), CD127 (20 μl; BD cat. no. 560549), CD4 (5 μl; BD cat. no. 562970), and CD3 (5 μl; BD cat. no. 563423) were added to the tubes as recommended by the manufacturer, whereas Foxp3 (5 μl; BD cat. no. 566526) was not added until the permeabilization process had been performed. 200 μl of whole heparinized blood was added to each tube. All tubes were vortexed briefly and incubated at room temperature in the dark for 30 min. Then, 2 ml of a 1:10 diluted Lyse solution from BD Biosciences was added to all tubes. Tubes were vortexed, incubated at room temperature in the dark for 10 min, and then were centrifuged at 1300 rpm for 8 min. Thereafter, supernatant was discarded, 1.5 ml PBS was added, and the tubes were vortexed again briefly. Then, the tubes were centrifuged at 1300 rpm for 8 min and the supernatant was discarded. 500 μl of 1:10 diluted BD Permeabilizing II solution was added to the tubes. After 10 min incubation, 1.5 ml PBS was added. Tubes were vortexed briefly and were subsequently centrifuged at 1300 rpm for 8 min. The supernatant was removed and discarded. Antibodies against the intracellular determinant Foxp3 were added to the pellets. After 30 min incubation in the dark at room temperature, 1.5 ml PBS was added. Tubes were vortexed again briefly and were subsequently centrifuged at 1300 rpm for 8 min. The supernatant was removed and discarded. Finally, 100 μl PBS was added to the pellets. Then the cells were analyzed. All samples were evaluated with eight-color fluorescence using the FACSCanto II triple-laser flow cytometer (BD Biosciences). At least 100,000 events were analyzed in the initial FSC/SSC dot plot. The gating strategy is depicted in Fig. 1 and supplementary files (1S and 2S).

### Determination of cytokines in plasma

IL-2, IL-4, IL-6, IL-10, IFN-γ, TNF-α (Magnetic Luminex® Performance Assay, Human High Sensitivity Cytokine Premixed Kit A; Lot number 1561687; R&D systems, Wiesbaden, Germany), and TGFβ1 (Magnetic Luminex® Performance Assay TGF-β1Base Kit; Lot number 175838; R&D systems, Wiesbaden, Germany) were determined in plasma according to the instructions of the manufacturer and were analyzed using the Luminex 200 system (Luminex, Austin, Texas, USA).

### Statistical analysis

Normally distributed data are presented as mean ± SD, whereas skewed data are presented as median and interquartile range (IQR). Spearman rank correlation test was conducted to show the association between different variables. Kruskal Wallis test with Dunn’s multiple comparison test was performed to show the differences between baseline characteristics as well as cell counts of different B cell subsets. In addition, multiple regression analysis with backward elimination was conducted to test whether CD19 + CD25+ Bregs are independently associated with eGFR or Tregs. Variables with *p*-values > 0.1 were excluded stepwise from the regression model. p-values ≤0.05 were considered significant. Finally, we converted GFR into a dichotomous variable (GFR < 30 vs. GFR ≥ 30 ml/min) to conduct a ROC analysis in order to analyze the sensitivity and specificity of the absolute counts of CD19+ CD25+ Bregs or Tregs, serum TGF-β1 (pg/ml), or serum IL-10 (pg/ml) in identifying a GFR ≥30 ml/min in renal transplant recipients (CKD stages 1-3 T according to KDIGO guidelines for evaluation and management of chronic kidney disease 2012) [27]. Statistical analysis was performed using IBM SPSS statistics 26 (IBM, Ehningen, Germany). *p*-values ≤0.05 were considered significant.

## Results

### CD19+ B cells and CD19 + CD25+ Bregs in healthy controls, ESKD patients and renal transplant recipients

**Fig. 2:**
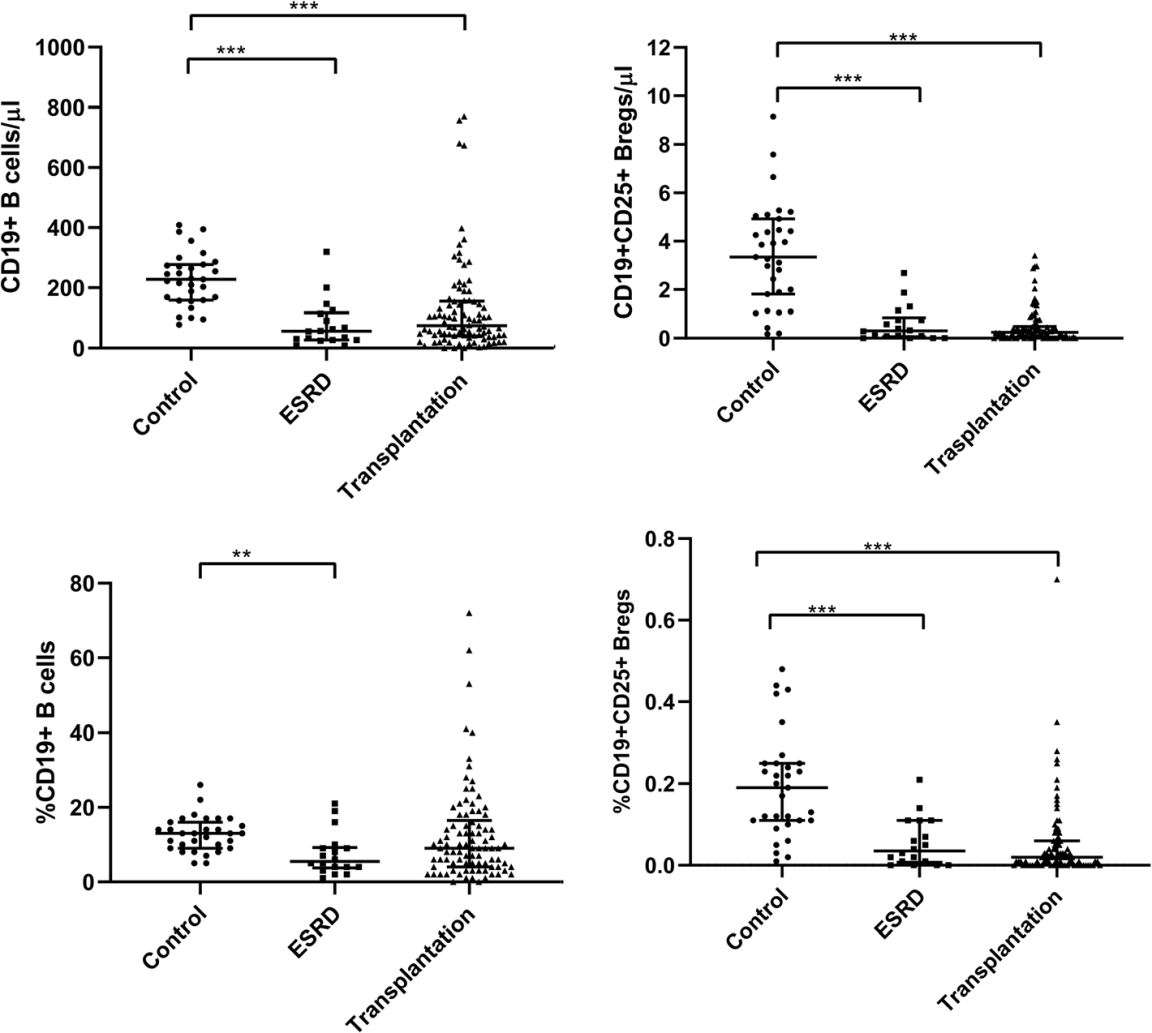
Absolute and relative CD19+ B cells and CD19 + CD25+ Bregs in healthy controls (**a**), ESKD patients (**b**), and renal transplant recipients (**c**). CD19+ B cells as well as CD19 + CD25+ Breg counts showed a marked decrease in ESKD and renal transplant patients**.** Adjusted *p* values are depicted (*p < 0.050, **p < 0.010, ***p < 0.001)

Compared with healthy controls, CD19+ B cells as well as CD19 + CD25+ Breg counts showed a marked decrease in ESKD patients (Fig. 2). A similar decrease was evident even after renal transplantation (Fig. 2). As expected, CD19 + CD25+ Bregs in healthy controls constituted < 2% of the CD19+ cell population. Despite their low counts in healthy controls, CD19 + CD25+ Bregs were significantly lower in ESKD patients and after renal transplantation. No significant difference could be demonstrated regarding the absolute or relative counts of CD19+ B cells or CD19 + CD25+ Bregs in ESKD patients compared to renal transplant recipients.

### Co-expression of Foxp3 on CD19 + CD25+ Bregs

To further characterize the CD19 + CD25+ Bregs, we analyzed the co-expression of Foxp3. As shown in Table 2, the majority of the CD19 + CD25+ Breg lacked expression of Foxp3.

**Table 2:** Comparison of the absolute and relative counts of CD19+ B cell subsets and CD4 + CD25 + CD127-Foxp3+ Tregs in healthy controls (HC), end-stage renal disease patients (ESRD) and renal transplant recipients (Tx)

	HC (***n*** = 31)	ESRD (***n*** = 18)	Tx (***n*** = 90)	***P***
**CD19+/μl**	228 (159–277)	56 (27–117)	74 (40–155)	0.009
**%CD19+**	13 (9–16)	5.5 (4–9)	9 (4–17)	< 0.001
**CD19 + CD25+/μl**	3.3 (1.8–5.2)	0.3 (0.06–0.8)	0.2 (0.04–0.47)	< 0.001
**%CD19 + CD25+**	0.2 (0.1–0.2)	0.03 (0.01–0.1)	0.02 (0.00–0.05)	< 0.001
**CD19 + CD25 + Foxp3+/μl**	0.16 (0.0–0.4)	0.0 (0.0–0.07)	0.0 (0.0–0.05)	< 0.001
**%CD19 + CD25 + Foxp3+**	0.01 (0.00–0.02)	0.00 (0.00–0.01)	0.00 (0.00–0.00)	< 0.001
**CD19 + CD25 + Foxp3−/μl**	3 (1.1–4)	0.23 (0.00–0.68)	0.2 (0.0–0.45)	< 0.001
**%CD19 + CD25 + Foxp3-**	0.17 (0.1–0.2)	0.02 (0.00–0.07)	0.02 (0.00–0.04)	< 0.001
**Treg/μl**	8 (4–16	4 (2–11)	2 (0.5–6)	< 0.001
**%Treg**	0.5 (0.2–0.7)	0.5 (0.3–1.2)	0.2 (0.1–0.5)	0.002

All values are expressed as median and Interquartile range % represent the proportion of cells to all lymphocytes (CD45+ cells)

### Etiology of ESKD in the renal transplantation patients and its association with the eGFR, CD19+ CD25+ Bregs, and Tregs

Chronic glomerulonephritis represented the most common cause of ESKD prior to transplantation in the renal transplant recipients in our study (36%). In 12 and 4.5% of the renal transplant recipients, the ESKD prior to renal transplantation was attributed to diabetes mellitus and hypertension, respectively. In 20% of the transplant recipients, ESKD occurred due to hereditary or congenital renal diseases (11% due to autosomal dominant polycystic kidney disease). The cause of ESKD prior to renal transplantation was unknown in 10% of the renal transplant recipients. Other causes of ESKD, such as obstructive uropathy, chronic pyelonephritis, and chronic interstitial nephritis were identified in 18% of the renal transplant recipients prior to renal transplantation. We compared the eGFR and the absolute counts of both CD19+ CD25+ Bregs and CD4 + CD25 + CD127- Foxp3+ Tregs among the different etiology groups. All different etiology groups showed no statistically significant difference regarding eGFR, CD19+ CD25+ Bregs, and Tregs (Table 3).

**Table 3:** Comparison of eGFR, CD19+ CD25+ Bregs, and Tregs among renal transplant recipients according to the etiology of ESKD prior to renal transplantation

	Chronic GN (***n*** = 32)	DN (***n*** = 11)	Hypertensive Nephropathy (***n*** = 4)	Hereditary or congenital (***n*** = 18)	Others ^a^ (***n*** = 16)	Unknown (***n*** = 9)	***P***-value
**eGFR (ml/min)**	38 (20–52)	43 (38–84)	22.5 (20–30)	46 (23–61)	30 (24–54)	39 (36–65)	0.20
**Tregs/**μl	1.7 (0.6–5)	1 (0.1–3)	1.8 (1–8)	1.3 (0.1–3.6)	3.7 (1–10)	1.2 (0.2–5)	0.19
**CD19+ CD25+ Bregs/**μl	0.2 (0–0.7)	0.2 (0–0.7)	0.2 (0–0.3)	0.3 (0.1–0.4)	0.4 (0–1.2)	0.2 (0–0.5)	0.86

*Abbreviations*: *eGFR* Estimated glomerular filtration rate, *GN* Glomerulonephritis, *DN* Diabetic nephropathy

^a^ Others include obstructive uropathy, chronic interstitial nephritis, and chronic pyelonephritis

### CD19 + CD25+ Bregs and standard immunosuppression after renal transplantation

**Fig. 3:**
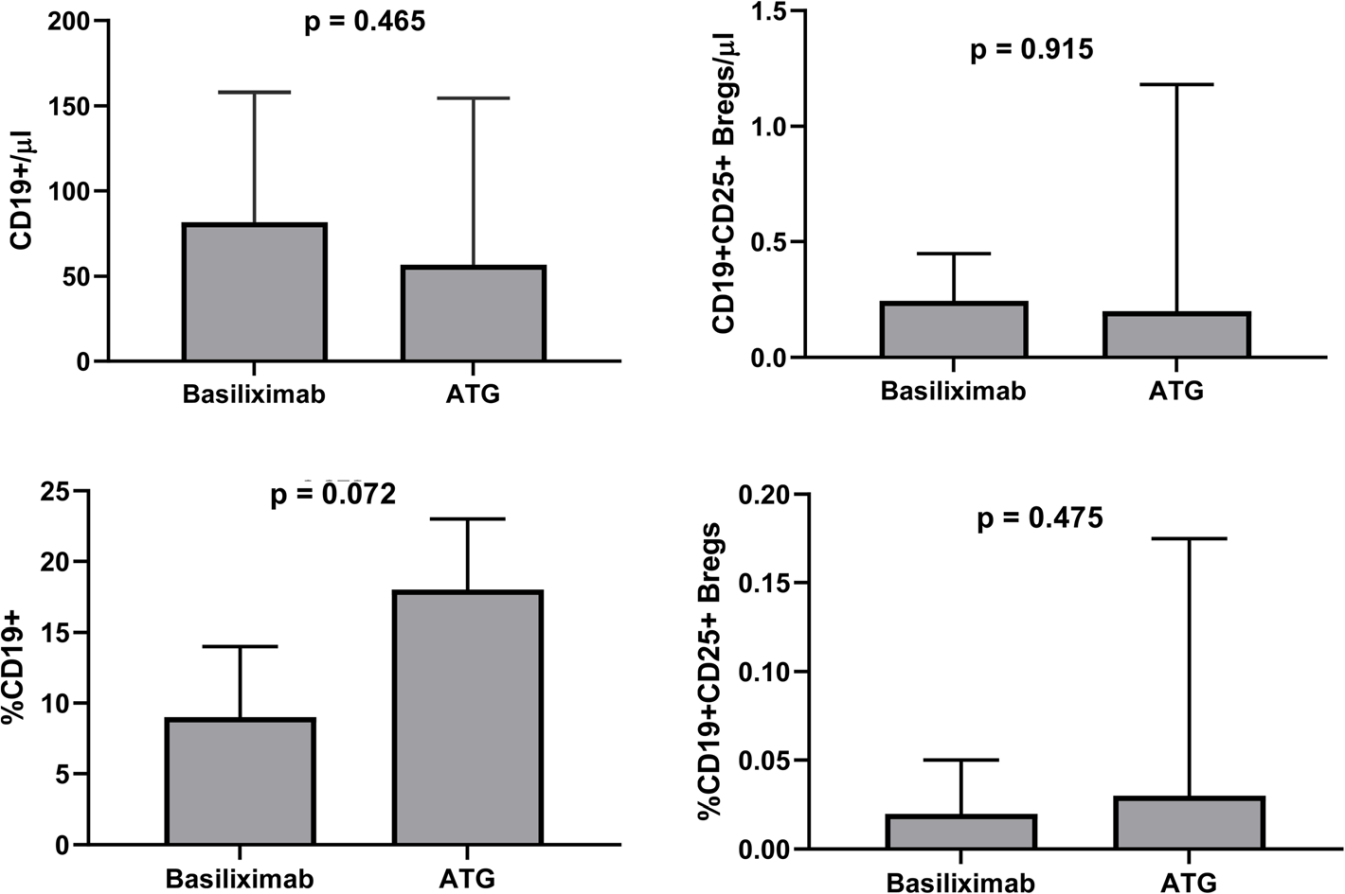
Comparison of absolute and relative counts of CD19+ B cells (**a** and **b**) and CD19+ CD25+ Bregs (**c** and **d**) between the Basiliximab-group and the ATG-group in renal transplant recipients. No statistically significant difference could be demonstrated between the two groups

**Fig. 4:**
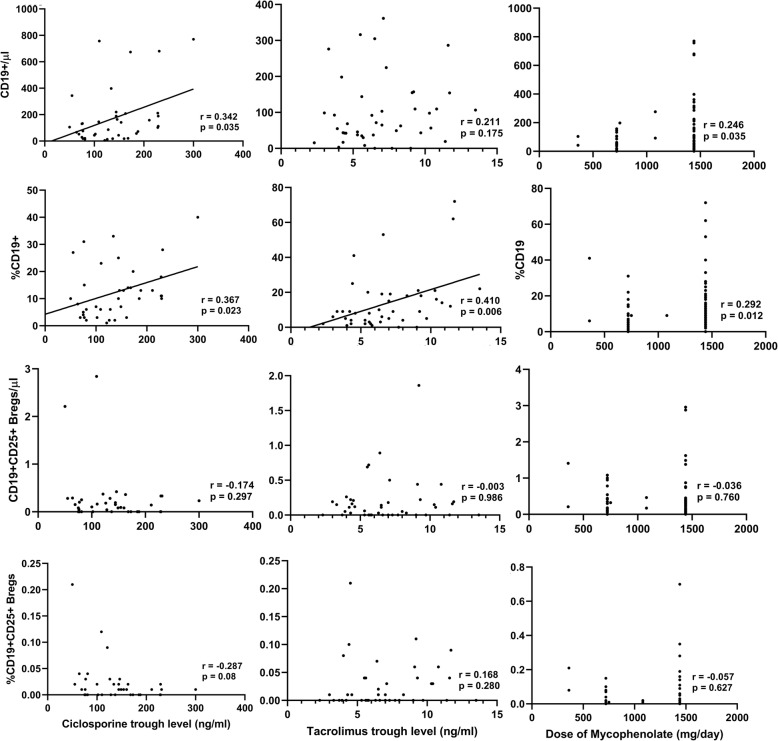
Correlation between the absolute or relative counts of CD19+ B cells or CD19 + CD25+ Bregs and ciclosporine (**a**) or tacrolimus trough levels (**b**), or the daily doses of mycophenolic acid in renal transplant recipients (**c**). A positive correlation was shown between ciclosporine trough levels and absolute CD19+ B cells but not CD19 + CD25+ Bregs in renal transplant recipients. Tacrolimus trough levels showed no correlation with absolute CD19 + B cells or CD19 + CD25+ Bregs. No significant correlation between mycophenolic acid daily doses and CD19 + CD25+ Bregs

The standard immunosuppression after renal transplantation consisted of induction and maintenance immunosuppression. For induction, basiliximab was used in the majority of cases, whereas anti-thymoglobulin (ATG) was reserved for high risk patients. Maintenance immunosuppression consisted of a combination of steroids, calcineurin-inhibitors (ciclosporine or tacrolimus), and anti-proliferative agents (mainly mycophenolic acid). As mentioned earlier, we found that the low counts of CD19+ B cells and CD19 + CD25+ Bregs in ESKD patients persisted after renal transplantation. In order to reveal whether the type of induction affected B cells differently, we compared the CD19+ B cells and CD19 + CD25+ Bregs between the group of patients who received basiliximab with those who received ATG. No statistically significant difference could be demonstrated (Fig. 3). Regarding maintenance immunosuppression, we found a positive correlation between ciclosporine trough levels and the absolute CD19+ B cell counts (r = 0.342, *p* = 0.035) but not the CD19 + CD25+ Bregs (r = − 0.174, *p* = 0.29) (Fig. 4). No correlation was found between tacrolimus trough levels and the absolute counts of CD19+ B cells or CD19 + CD25+ Bregs. Likewise, we found no significant correlation between mycophenolic acid daily doses and the CD19 + CD25+ Bregs (Fig. 4).

### Association between CD19+ B cells or CD19 + CD25+ Bregs and IL-10, TGF-β1, and INF-γ

A significant negative correlation between the absolute counts or the percentages of CD19+ B cells and serum IFN-γ (r = 0.346, *p* = 0.001; r = 0.364, *p* < 0.001, respectively) was shown in renal transplant recipients (Tables 4 and 5). Moreover, we found a negative correlation between the absolute count of CD19+ B cells and serum IL-10 (r = 0.299, *p* = 0.004) (Table 5).

**Table 4:** Association of relative CD19+ B cells or CD19 + CD25+ Bregs counts with IL-10, TGF-β1, and IFN-γ in healthy controls (HC), end-stage renal disease patients (ESRD) or renal transplant recipients (Tx)

	HC				ESRD				Tx			
	%CD19		%CD19 + CD25+		%CD19		%CD19 + CD25+		%CD19		%CD19 + CD25+	
	***r***	***p***	***r***	***p***	***r***	***p***	***r***	***p***	***r***	***p***	***r***	***p***
**IL-10 (pg/ml)**	0.114	0.541	−0.027	0.887	−0.184	0.465	− 0.275	0.269	− 0.174	0.102	− 0.025	0.818
**TGF-β1 (pg/ml)**	0.115	0.536	0.084	0.654	−0.386	0.113	− 0.121	0.631	−0.015	0.891	0.167	0.115
**IFN-γ (pg/ml)**	−0.199	0.283	0.202	0.276	0.189	0.454	−0.099	.697	**−0.364**	**< 0.001**	0.145	0.176

**Table 5:** Association of the absolute CD19+ B cells and CD19 + CD25+ Bregs count with IL-10, TGF-β1 and IFN-γ in healthy controls (HC), end-stage renal disease patients (ESRD) and renal transplant recipients (Tx)

	HC				ESRD				Tx			
	CD19/μl		CD19 + CD25+/μl		CD19/μl		CD19 + CD25+/μl		CD19/μl		CD19 + CD25+/μl	
	*r*	*p*	*r*	*p*	*r*	*p*	*r*	*p*	*r*	*p*	*r*	*p*
**IL-10 (pg/ml)**	0.082	0.662	−0.070	0.708	−0.210	0.404	−0.283	0.255	**−0.299**	**0.004**	−0.121	0.258
**TGF-β1 (pg/ml)**	0.039	0.835	0.006	0.973	−0.243	0.332	−0.146	0.562	0.155	0.144	**0.255**	**0.015**
**IFN-γ (pg/ml)**	−0.126	0.500	0.170	0.361	0.102	0.687	−0.119	0.637	**−0.346**	**0.001**	0.055	0.606

No significant correlation between the absolute counts or the percentage of CD19 + CD25+ Bregs and TGF-β1 or IFN-γ was found in healthy controls. Likewise, we found no correlation between these serum cytokine levels and CD19 + CD25+ Bregs in ESKD patients (Tables 4 and 5). In contrast, a positive correlation between the absolute count, but not the percentage, of CD19 + CD25+ Bregs and TGF-β1 was shown in renal transplant recipients (r = 0.255, *p* = 0.015) (Table 5).

### Association between CD19+ CD25+ Bregs or CD4+ CD25+ CD127- Foxp3+ Tregs and serum CRP (mg/l), IL-6 (pg/ml), or TNF-α (pg/ml) in ESKD patients and renal transplant recipients

To reveal whether an inflammatory milieu might have affected the absolute counts of CD19 + CD25+ Bregs and Tregs in the ESKD and renal transplant patients, we conducted a correlation analysis between the CD19+ CD25+ Bregs or CD4 + CD25 + CD127-Foxp3 + Tregs and serum CRP, IL-6, or TNF-α in both of these patient populations. Neither in the ESKD nor in the renal transplant patients could a significant correlation be found between the absolute counts of either CD19+ CD25+ Bregs or Tregs and serum CRP, IL-6, or TNF-α (Table 6).

**Table 6:** Correlation between Tregs or CD19+ CD25+ Bregs and CRP, IL-6, TNF-α in ESKD patients and renal transplant recipients

	ESKD						RTX					
	CRP (mg/l)		IL6(pg/ml)		TNF-α (pg/ml)		CRP (mg/l)		IL6(pg/ml)		TNF-α (pg/ml)	
	***r***	***p***-	***r***	***p***-	***r***	***p***-	***r***	***p***-	***r***	***p***-	***r***	***p***-
**Tregs/μl**	−0.2	0.32	0.36	0.88	−0.2	0.37	−0.06	0.56	−0.1	0.31	−0.1	0.31
**CD19 + CD25+ Bregs/μl**	−013	0.59	0.10	0.68	0.14	0.56	−0.07	0.47	−0.2	0.054	−0.1	0.41

### Correlation between CD19 + CD25+ Bregs and CD4 + CD25 + CD127-Foxp3+ Tregs

**Fig. 5:**
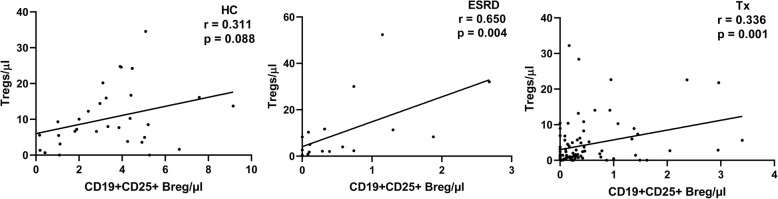
Association of the absolute counts of Tregs with the absolute counts of CD19 + CD25+ cells in healthy controls (HC) (**a**), end-stage kidney disease patients (ESKD) (**b**), and renal transplant recipients (Tx) (**c**). A significant positive correlation was shown in ESKD patients as well as renal transplant recipients

We found a significant positive correlation between the absolute counts of CD19 + CD25+ Bregs and CD4 + CD25 + CD127-Foxp3+ Tregs in ESKD patients as well as renal transplant recipients (*r* = 0.650, *p* = 0.004; *r* = 0.336, *p* = 0.001, respectively) (Fig. 5). This suggests that the regulatory function of CD19 + CD25+ Bregs might be mediated through activation of Tregs. Although a trend for positive correlation between the absolute counts of those 2 cell subsets was shown in healthy controls, it did not reach statistical significance (*r* = 0.311, *p* = 0.08).

In order to show whether CD19 + CD25+ Bregs were independently associated with Tregs in renal transplant recipients, we conducted a backward multiple linear regression analysis to adjust for possible confounders (CD4+ T cells, CD8+ T cells, CD16 + CD56+ NK cells, CD19+ B cells, IL-2, IL-4, IL-6, IL-10, IFN-γ, TNF-α, and TGF-β1). CD19 + CD25+ Bregs were independently positively associated with Tregs (unstandardized B = 2.8, 95% CI (0.9–4.6), p = 0.004) (Table 7).

**Table 7:** Multiple linear regression analysis with backward elimination method of the factors affecting Treg counts in renal transplant recipients

Variables	Unstandardized B (95% CI) ***n*** = 75	Standardized beta	***p***	
CD4+/μl	0.003 (−0.001–0.006)	0.17	0.12	excluded
CD8+/μl	0.003 (−0.004–0.009)	0.10	0.42	excluded
CD19+/μl	−0.006 (− 0.2–0.006)	−0.13	0.31	excluded
CD16 + CD56+/μl	−0.003 (− 0.02–0.01)	−0.05	0.60	excluded
CD19 + CD25+/μl	3 (1–4.6)	0.3	**0.004**	
IL-2 (pg/ml)	−0.4 (−1–0.1)	−0.15	0.14	excluded
IL-4 (pg/ml)	−0.1 (− 0.2–0.05)	−0.13	0.19	excluded
IL-6 (pg/ml)	0.001 (−0.004–0.005)	0.04	0.74	excluded
IL-10 (pg/ml)	−0.02 (− 0.1–0.05)	−0.1	0.61	excluded
TGF-ß1 (pg/ml)	0	0.03	0.76	excluded
TNF-α (pg/ml)	−0.03 (−0.2–0.1)	−0.05	0.65	excluded
IFN γ (pg/ml)	0.03 (−0.1–0.1)	0.08	0.51	excluded

### Association between CD19 + CD25+ Bregs and eGFR in renal transplant recipients

We analyzed the correlation between the absolute count of CD19 + CD25+ Bregs and eGFR, assessed with the CKD-EPI formula, in renal transplant recipients. A significant positive correlation was shown. We found no significant association between the eGFR and tacrolimus or ciclosporin trough levels in renal transplant recipients (data not shown). In order to show whether CD19 + CD25+ Bregs and their subpopulations are independently associated with eGFR in renal transplant recipients, we conducted a multiple regression analysis using backward elimination to adjust for potential confounders, including age, type of donor (living vs. deceased donor), cold ischemia time, induction immunosuppression (basiliximab vs. ATG), delayed graft function, days post-transplantation, HLA mismatches > 3 (A, B, DR), previous CMV infection or BK-nephropathy, previous rejection, proteinuria measured with the protein/creatinine ratio, daily steroid dose, Treg counts, and CRP. CD19 + CD25+ Bregs remained significantly positively associated with eGFR (unstandardized B = 11, 95% CI (1.5–20), *p* = 0.02). As expected, renal grafts from living donors showed better eGFR than deceased donor grafts (unstandardized B = 15, 95% CI (0.1–29), *p* = 0.049) (Table 8).

**Table 8:** Multiple linear regression analysis with backward elimination method of the factors affecting estimated GFR in renal transplant recipients

Variables	Unstandardized B (95% CI) ***n*** = 75	Standardized beta	***p***	
Age	−0.002 (− 0.6–0.5)	−0.001	0.99	excluded
Days post-transplantation	0.0 (−0.004–0.004)	0	1.000	excluded
Living vs deceased donor	15 (0.1–29)	0.22	**0.049**	
Cold ischemia time (hours)	0.15 (−2–2)	0.03	0.87	excluded
HLA-mismatches (HLA-A, B, DR) > 3	0.8 (−13–14)	0.014	0.9	excluded
Delayed graft function	−2 (− 26–22)	0.02	0.88	excluded
Induction (Basiliximab vs. ATG)	13 (−1–26)	0.2	0.07	excluded
Previous rejections	−5 (−19–9)	−0.1	0.46	excluded
Daily steroid dose (mg)	−0.4 (−1–0.5)	−0.1	0.40	excluded
Protein-creatinine-ratio (g/mol)	−0.02 (− 0.1–0.03)	−0.1	0.31	excluded
Previous CMV	3 (− 11–18)	0.05	0.68	excluded
Previous BKVN	−19 (−49–11)	−0.15	0.21	excluded
CRP (mg/l)	−0.1 (− 0.2–0.02)	−0.2	0.053	excluded
CD19 + CD25+ Bregs/μl	11 (1.5–20)	0.26	**0.02**	
Tregs/μl	−0.4 (−1.5–0.4)	−0.1	0.34	excluded

### ROC analysis to identify sensitivity and specificity of absolute counts of CD19 + CD25+ Bregs or Tregs, serum TGF-β1, or serum IL-10 in identifying CKD (chronic kidney disease) stage 1-3 T in renal transplant recipients

**Fig. 6:**
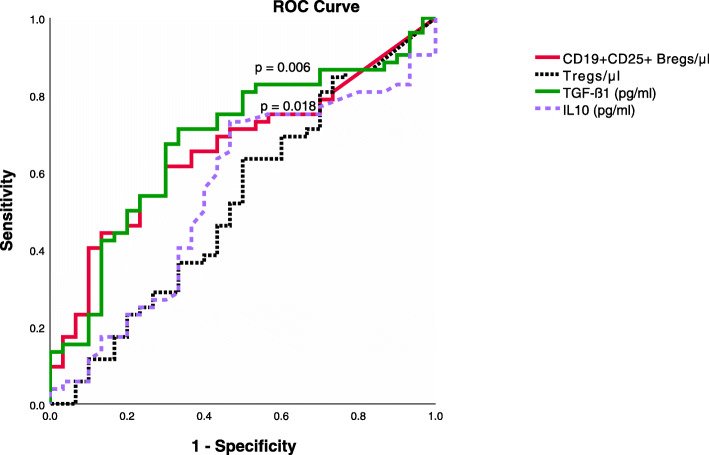
Area under the curves (AUCs) of receiving operator characteristics for the absolute counts of CD19 + CD25 + Bregs and Tregs, serum TGF-ß1, and serum IL-10 as possible identifiers of eGFR ≥ 30 ml/min. The optimal cutoff values were obtained through analysis of coordinate points of ROC curve. The AUC of CD19 + CD25 + Bregs as well as serum TGFβ1 showed statistical significance (AUC = 0.66, *p* = 0.018, AUC = 0.68, *p* = 0.006, respectively). AUC values of Treg absolute count as well as serum IL-10 were not significantly increased (AUC = 0.52, *p* = 0.7; AUC = 0.54, *p* = 0.5, respectively)

We categorized the GFR in renal transplant recipients into 2 groups: GFR < 30 ml/min and GFR ≥ 30 ml/min. A cut-off of GFR of 30 ml/min was selected, based on the observation that renal transplant recipients with GFR below 30 ml/min (CKD stage 4-5 T) were more vulnerable to a rapid decline of renal function and graft loss [28]. ROC analysis was performed to analyze sensitivity and specificity of the absolute counts of CD19+ CD25+ Bregs or Tregs, serum TGF-ß1 (pg/ml), or serum IL-10 (pg/ml) in identifying a GFR ≥30 ml/min. From the ROC curve coordinate points, we deem a cut-off of an absolute CD19 + CD25+ Breg count of 0.12 cell/μl (AUC = 0.66, *p* = 0.018, sensitivity 75%, specificity 43%, positive predictive value 70%, and negative predictive value 46%) and a cut-off of serum TGF-ß1 of 19,635.4 pg/ml (AUC = 0.68, *p* = 0.006, sensitivity 81%, specificity 50%, positive predictive value 74%, and negative predictive value 36%) to be best for predicting a GFR ≥ 30 ml/min. In contrast, AUC values of Treg absolute count as well as serum IL-10 were not useful identifiers of CKD stages 1-3 T (AUC = 0.52, *p* = 0.7; AUC = 0.54, *p* = 0.5, respectively) (Fig. 6).

## Discussion

The regulatory role of B cells has gained importance during the last decade due to their potential role in operational immune tolerance. The identification of B cell subsets with regulatory functions might open the way to promising therapeutic strategies in the field of transplantation aiming to reduce the dose of immunosuppressive drugs and to prolong the graft survival. In contrast to Tregs, which exhibit a characteristic established phenotype, Bregs show different phenotypes and exert their immunosuppressive function through IL-10-dependent as well as IL-10-independent mechanisms. CD19 + CD25+ Bregs have been shown to possess immunoregulatory function. An in vitro study showed that CD19 + CD25 + Bregs exert a dose-dependent inhibition of CD4+ T cells and a stimulation of Foxp3 and CTLA-4 expression in Tregs [26]. Although the mechanisms, through which CD19 + CD25+ Bregs mediate their function, have been shown in various studies, the role of CD19 + CD25+ Bregs has not yet been studied in renal transplant recipients in terms of a potential contribution to a better GFR. In the current study we aimed primarily to show a potential impact of CD19 + CD25+ Bregs on eGFR in renal transplant recipients. As secondary objectives, we provide statistical evidence of a potential interaction between CD19 + CD25 + Bregs and Tregs in renal transplant recipients. In addition, we aimed to further characterize the CD19 + CD25 + Bregs through their expression of Foxp3.

We showed that CD19 + CD25+ Bregs are significantly higher in healthy controls than in patients with ESKD or transplant recipients. The potential role of Bregs in renal transplant recipients can be understood through the observation of Clatworthy et al., who reported an increased incidence of acute cellular rejection in renal transplant recipients, in whom the induction was conducted with rituximab. This might be attributable to a depletion of Bregs [29]. In contrast, renal transplant recipients with operational tolerance demonstrated increased numbers of total B cells, memory B cells, and transitional B cells, compared to patients with chronic rejection or stable long-term renal function under standard immunosuppression. Interestingly, the same study showed also an increased BAFF-R/BAFF ratio in the immunosuppression-free transplant recipients, a factor that might contribute to the increase in the total counts of B cells in this patient population [30]. Although our study showed better renal function with higher CD19 + CD25+ Breg counts in renal transplant recipients, the CD19 + CD25+ Bregs in those patients were significantly lower than in healthy controls, suggesting a possible negative impact of immunosuppression on Bregs. In the present study, however, we found no association between CD19 + CD25+ Breg counts and tacrolimus or ciclosporine trough levels. Furthermore, we found no correlation between steroid or mycophenolate doses and CD19 + CD25+ Breg counts. Intriguingly, both ESKD patients and renal transplant recipients showed low B cell counts. Although the decrease in B cell counts in ESKD patients might be attributed to decreased BAFF receptors on transitional B cells [31, 32], other mechanisms seem to play a role in renal transplant recipients. Whereas calcineurin inhibitors (CNI) marginally inhibited B cells in vitro dependent of the degree of B cell activation, mycophenolic acid as well as rapamycin suppressed B cell responses significantly [33]. In the current study, we found no supporting evidence of a possible effect of inflammatory milieu on the absolute counts of CD19+ CD25+ Bregs and Tregs in the view of absence of a significant correlation between the absolute counts of either CD19 + CD25 + Bregs or CD4 + CD25 + CD127-Foxp3 + Tregs and serum CRP, IL-6, or TNF-α in both ESKD and renal transplant recipients.

Literature reports show similarities between Tregs and Bregs. Like Tregs, Bregs can express IL-10, TGF-ß, and/or Foxp3. Intriguingly, it was shown that Bregs predominate earlier in the inflammatory response, apparently enhancing the appearance of Tregs, and disappear when Tregs operate [34]. In accordance with this, we found that CD19 + CD25+ Bregs were positively correlated with CD4 + CD25 + CD127-Foxp3+ Tregs in ESKD patients as well as in transplant recipients. In addition, in our regression model, Bregs remained independently positively associated with Tregs in renal transplant recipients after adjustment for other confounding factors.

In a study conducted by Kessel et al., it was shown that the regulatory function of Bregs on Tregs was independent of IL-10 [26]. Supporting this finding is our observation of an absence of association between the absolute counts of CD19 + CD25+ cells and serum IL-10 in healthy controls, ESKD patients, and renal transplant recipients. CD19 + CD25+ Bregs were shown to enhance Foxp3 and CTLA-4 expression in Tregs through direct contact [26]. Recently, and as another possible mechanism of action, a study has shown that CD19 + CD25+ Bregs in allergic rhinitis patients were lower and showed lower expression of PD-L1 compared with healthy controls. In addition, anti-PD-L1 enhanced CD19 + CD25+ Breg apoptosis and significantly decreased IL-10 expression [35]. Whether the expression of CD25 on B cells would be associated with local depletion of IL-2 and hence deprivation of effector T cells from activation signals leading to their apoptosis remains intriguing but speculative.

Interferon gamma (IFN-γ) represents a cytokine with paradoxical functions. Whereas it is capable of triggering Th1-mediated immune responses, it can also exert regulatory functions. It has been reported that IFN-γ expression by Tregs is of a paramount importance to their function in vivo [36]. The early production of IFN-γ by stimulated Tregs suppresses the activation and proliferation of the T cells that express IFN-γR1 and IFN-γR2 [37]. Paradoxical to the regulatory effect of IFN-γ expression in Tregs, IFN-γ-expressing B cells, termed B1 cells, suppress Tregs and induce inflammatory responses [38]. In the current study, CD19+ B cells were negatively correlated with the serum IFN-γ levels in the renal transplant recipients. In contrast, CD19+ CD25+ Bregs showed no correlation with the serum IFN-γ levels in the same patient group.

Notably, the majority of CD19 + CD25+ Bregs in our study did not express Foxp3. Since the majority of our study patients represented clinically stable patients with no major inflammatory responses, it might be intuitive that most of the CD19 + CD25+ Bregs represented resting Bregs. This finding is in line with that of Vadasz et al., who found that resting CD19 + CD25+ Bregs in SLE patients lacked significant expression of Foxp3. After stimulation of these cells with CpG-ODN and CD40L, marked expression of Foxp3 was noted. In addition, the regulatory effect of Bregs on Tregs was partially mediated through TGF-β [26]. In accordance with this finding, we found a positive correlation between CD19 + CD25+ Bregs and serum TGF-β1 in renal transplant recipients. Moreover, our ROC analysis showed serum TGF-ß1 as a useful identifier of CKD stages 1-3 T.

To show whether an increase in CD19 + CD25+ Breg counts translates to an increase in GFR, we conducted a multiple linear regression analysis to adjust for confounders. We found that CD19 + CD25+ Bregs are independently positively associated with GFR. This finding supports the in vitro proven regulatory function of CD25+ Bregs and reveals the potential importance of CD25+ Bregs in renal transplant recipients as indicated by their association with better graft function. Although CD19 + CD25 + Bregs independently correlated with both eGFR and absolute peripheral Treg counts, Tregs showed no significant association with eGFR, suggesting that CD19 + CD25+ Bregs affect the renal graft function through multiple mechanisms dependent and independent of Tregs. Finally, this study identified absolute CD19 + CD25+ Breg counts and serum TGF-ß1 levels above a cut-off of 0.12/μl and 19,635.4 pg/ml, respectively, to be the best at identifying CKD stages 1–3 T (GFR ≥ 30 ml/min). In contrast, absolute Tregs as well as serum IL-10 were not useful identifiers of CKD stages 1-3 T. To our knowledge, this is the first study to show the potential positive impact of CD19 + CD25 + Bregs on graft function in renal transplant recipients.

In summary, our findings indicate that higher counts of CD25+ Bregs are independently associated with better renal function and higher absolute Treg counts in renal transplant recipients. This finding might open the door for further assessment of the regulatory potential of CD19 + CD25 + Bregs in randomized controlled studies hoping for identifying promising therapeutic strategies as a further step on the way to improve renal graft longevity in renal transplant recipients.

### Limitations of the study

To reveal the exact mechanisms by which CD19 + CD25+ cells exert their function in renal transplant recipients, in vitro experiments need to be conducted. In order to confirm an in vivo effect of CD19 + CD25 + Bregs on renal graft function, randomized controlled trials should be conducted.

## Supplementary Information

**Additional file 1.**

## Data Availability

The datasets used and/or analyzed during the current study are available from the corresponding author on reasonable request.

## Abbreviations

ATG: Antithymocyte globulin
BAFF: B cell activating factor
Bregs: Regulatory B-cells
CNI: Calcineurin inhibitors
CTLA-4: Cytotoxic T-lymphocyte-associated Protein 4
eGFR: estimated glomerular filtration rate
ESKD: End-stage kidney disease
Foxp3: Forkhead Box p3
IFN-γ: Interferon gamma
IL-10: Interleukin 10
NK: Natural killer cells
TGF-ß: Transforming growth factor beta
TNF-α: Tumor necrosis factor alpha
Tregs: Regulatory T cells
