# Supplementary material for: Higher CD19+CD25+ Bregs are independently associated with better graft function in renal transplant recipients
Source: BMC Nephrol. 2021 May 17;22:180. doi: 10.1186/s12882-021-02374-2 (PMC8127305; doi:10.1186/s12882-021-02374-2)
Supplement: Supplementary file 1 — Additional file 1. [file 12882_2021_2374_MOESM1_ESM.docx]

**6**

**Healthy control**

**RTX Patient**

**Figure 1S**

**RTX Patient**

**Healthy Control**

**2**

**4**

**6**

**1**

**3**

**5**

**Figure 2S**
